# Obesity and survival in critically ill patients with acute respiratory distress syndrome: a paradox within the paradox

**DOI:** 10.1186/s13054-017-1682-5

**Published:** 2017-05-03

**Authors:** Lorenzo Ball, Ary Serpa Neto, Paolo Pelosi

**Affiliations:** 1 IRCCS AOU San Martino-IST, Department of Surgical Sciences and Integrated Diagnostics, University of Genoa, Largo Rosanna Benzi 8, 16131 Genoa, Italy; 2 Department of Critical Care Medicine, Hospital Israelita Albert Einstein, São Paulo, Brazil

**Keywords:** ARDS, Obesity, ICU

The incidence of obesity is steadily increasing, and its prevalence, defined as a body mass index (BMI) above 30 kg/m^2^, is 13% in the world adult population, and rises to up to 40% in high-income countries [1]. As a consequence, around 20% of the patients admitted to the intensive care unit (ICU) are obese [2]. Obesity and overweight are associated with an increased risk of death in the general population [3], but in specific disease conditions a decrease in mortality has been reported: this is the case of patients with septic shock [4] and acute respiratory distress syndrome (ARDS), and is referred to as the *obesity paradox*. The association between higher BMI and lower mortality is difficult to interpret and potentially influenced by several confounding factors. In patients with ARDS, this paradox is particularly surprising, as obese patients have peculiar alterations of the respiratory function, such as increased chest wall elastance and lower total respiratory system compliance, posing specific challenges for the clinician when mechanical ventilation is required [5, 6].

The obesity paradox in ARDS patients has been investigated in several studies and two recent meta-analyses [7, 8]. Ni and co-authors [7] analysed the evidence concerning the association between BMI and clinical outcomes in ARDS patients, pooling data from 6268 patients enrolled in five studies, including three prospective observational studies [9–11], a retrospective cohort study [12] and one randomised controlled trial [13]. The authors conclude that obesity and morbid obesity were associated with a lower mortality rate in patients with ARDS, therefore supporting the concept of the *obesity paradox*. In another recent analysis including four additional studies, Zhi et al. [8] reported that obesity increased ARDS-associated morbidity in the ICU population; however, mortality due to ARDS in obese was lower compared to non-obese patients. An increased mortality among underweight patients was also reported, which could be explained by the worse clinical conditions and comorbidities of patients admitted to the ICU with impaired nutritional status.

In the five studies included by Ni et al., [7] obese patients were systematically younger and had lower severity scores compared to the reference group (normal weight), while the opposite was observed in the underweight patients. The analysis by Zhi et al*.* [8] does not report patients’ severity scores. Figure 1 illustrates the observed unadjusted mortality rates in the different obesity classes for the studies included in the meta-analyses for which severity scores (SAPS II predicted mortality or APACHE III) could be extracted: the trend in mortality in the different obesity classes is similar to that of illness severity. This could suggest that the effect of BMI on mortality might be mediated by other clinical factors.

**Fig. 1:**
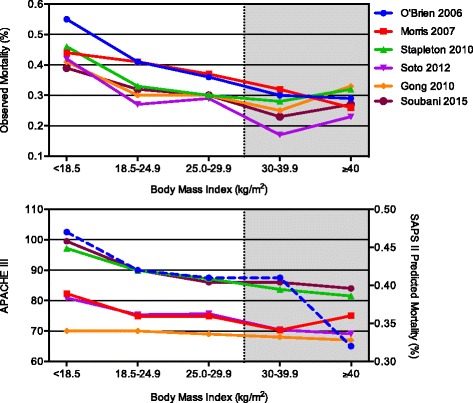
Mortality and disease severity in different BMI classes. *Upper panel*: unadjusted observed mortality rates in the five BMI classes. *Lower panel*: APACHE III severity scores. For one study [9] the APACHE III score was not reported; therefore, the SAPS II predicted mortality is plotted (*blue dashed line*). This figure reports data from only the studies included in the two meta-analyses [14, 15] for which disease severity was available. O’Brien 2006 [9], Morris 2007 [11], Stapleton 2010 [13], Soto 2012 [12], Gong 2010 [10], Soubani 2015 [22]

Both meta-analyses present some limitations: 1) inclusion of retrospective studies; 2) lack of adjustment for confounders; 3) most analyses are restricted to studies in which obesity was classified according to the WHO BMI classes; and 4) the presence of heterogeneity in some analyses. The adjustment for potential confounding factors is virtually impossible in a conventional meta-analysis without access to individual patient data, especially when a limited number of studies is included [14]. Indeed, when correcting for confounders, BMI was no longer associated with mortality in one of the studies [10], as previously reported in mechanically ventilated patients [15].

Several pathophysiological mechanisms and clinical management-related factors could explain the decreased mortality in obese critically ill patients, including those with ARDS. Recent evidence suggests the existence of a protective response called *pre-conditioning cloud* where obesity induces a low-grade inflammation, generating a process that subsequently protects the lung against further insults [16]. Indeed, *pre-conditioning* implies that a chronic pro-inflammatory status creates a protective environment, limiting the detrimental effects of a more aggressive *second hit*, such as ventilator-induced lung injury or sepsis. Obesity itself increases the plasma and adipose levels of inflammatory cytokines, including in ARDS [13], and the adipose-triggered inflammatory mediators could alter peripherally the physiological responses to injury, contributing to abnormalities in systemic and pulmonary circulation [17]. Some of these mechanisms are similar to the endogenous reactive protection occurring during endotoxemia in healthy subjects [18]. Also, during critical illness adipose tissue macrophages shift from pro-inflammatory M1 to alternative or anti-inflammatory M2 phenotypes [19]. Finally, the concept of *metabolically healthy obesity* (MHO) has been recently proposed, referring to obese individuals without associated metabolic comorbidities [20]. Interestingly, MHO has been linked to weaker adipose-related inflammatory activity and lower mortality risk compared to individuals with metabolically unhealthy obesity [20]. Moreover, clinicians tend to consider obese patients at high risk of worse outcome; thus, this might result in earlier admission to the ICU for monitoring purposes as well as increased use of prophylactic measures such as early mobilisation, more cautious pressure ulcer prevention, stricter glycaemic control, and more attention paid to mechanical ventilation parameters [21]. Moreover, in obesity, the high chest wall elastance could redistribute regional transpulmonary pressure, possibly reducing the potential negative effects of mechanical ventilation in an inhomogeneous lung.

Meta-analyses of observational studies are a tool for generating experimental hypotheses to be tested in other experimental settings, and their results should be cautiously interpreted as an association, not necessarily implying causality. It would be extremely important to answer the question of whether the *obesity paradox* in ARDS is mediated by other factors: if the association is confirmed it could open new perspectives for the management of respiratory failure. A quasi-experimental setting could be achieved by performing an individual data meta-analysis, but further physiological and clinical studies are warranted to better understand the interaction between obesity and response to critical illness.

## Abbreviations

APACHE: Acute Physiology and Chronic Health Evaluation
ARDS: Acute respiratory distress syndrome
BMI: Body mass index
ICU: Intensive care unit
MHO: Metabolically healthy obesity
SAPS: Simplified Acute Physiology Score
WHO: World Health Organization.

## References

[1] Flegal KM, Kruszon-Moran D, Carroll MD, Fryar CD, Ogden CL (2016). Trends in obesity among adults in the United States, 2005 to 2014. JAMA..

[2] Lewandowski K, Lewandowski M (2011). Intensive care in the obese. Best Pract Res Clin Anaesthesiol..

[3] Adams KF, Schatzkin A, Harris TB, Kipnis V, Mouw T, Ballard-Barbash R (2006). Overweight, obesity, and mortality in a large prospective cohort of persons 50 to 71 years old. N Engl J Med..

[4] Arabi YM, Dara SI, Tamim HM, Rishu AH, Bouchama A, Khedr MK (2013). Clinical characteristics, sepsis interventions and outcomes in the obese patients with septic shock: an international multicenter cohort study. Crit Care Lond Engl.

[5] Ball L, Sutherasan Y, Pelosi P (2013). Monitoring respiration: what the clinician needs to know. Best Pract Res Clin Anaesthesiol..

[6] Pelosi P, Gregoretti C (2010). Perioperative management of obese patients. Best Pract Res Clin Anaesthesiol..

[7] Ni Y-N, Luo J, Yu H, Wang Y-W, Hu Y-H, Liu D (2017). Can body mass index predict clinical outcomes for patients with acute lung injury/acute respiratory distress syndrome? A meta-analysis. Crit Care Lond Engl..

[8] Zhi G, Xin W, Ying W, Guohong X, Shuying L (2016). “Obesity paradox” in acute respiratory distress syndrome: a systematic review and meta-analysis. PLoS One..

[9] O’Brien JM, Phillips GS, Ali NA, Lucarelli M, Marsh CB, Lemeshow S (2006). Body mass index is independently associated with hospital mortality in mechanically ventilated adults with acute lung injury. Crit Care Med..

[10] Gong MN, Bajwa EK, Thompson BT, Christiani DC (2010). Body mass index is associated with the development of acute respiratory distress syndrome. Thorax..

[11] Morris AE, Stapleton RD, Rubenfeld GD, Hudson LD, Caldwell E, Steinberg KP (2007). The association between body mass index and clinical outcomes in acute lung injury. Chest..

[12] Soto GJ, Frank AJ, Christiani DC, Gong MN (2012). Body mass index and acute kidney injury in the acute respiratory distress syndrome. Crit Care Med..

[13] Stapleton RD, Dixon AE, Parsons PE, Ware LB, Suratt BT, Acute Respiratory Distress Syndrome Network NHLBI (2010). The association between BMI and plasma cytokine levels in patients with acute lung injury. Chest..

[14] Egger M, Schneider M, Davey SG (1998). Spurious precision? Meta-analysis of observational studies. BMJ..

[15] Anzueto A, Frutos-Vivar F, Esteban A, Bensalami N, Marks D, Raymondos K (2011). Influence of body mass index on outcome of the mechanically ventilated patients. Thorax..

[16] Bustamante AF. Adipose-lung cell crosstalk in the obesity-ARDS paradox. J Pulm Respir Med. 2013;3:144. doi:10.4172/2161-105X.

[17] Teoh N (2003). Low-dose TNF-α protects against hepatic ischemia-reperfusion injury in mice: Implications for preconditioning. Hepatology..

[18] Shah R, Hinkle CC, Haris L, Shah R, Mehta NN, Putt ME (2012). Adipose genes down-regulated during experimental endotoxemia are also suppressed in obesity. J Clin Endocrinol Metab..

[19] Langouche L, Marques MB, Ingels C, Gunst J, Derde S, Vander Perre S (2011). Critical illness induces alternative activation of M2 macrophages in adipose tissue. Crit Care..

[20] Durward CM, Hartman TJ, Nickols-Richardson SM (2012). All-cause mortality risk of metabolically healthy obese individuals in NHANES III. J Obes..

[21] Pompilio CE, Pelosi P, Castro MG (2016). The bariatric patient in the intensive care unit: pitfalls and management. Curr Atheroscler Rep..

[22] Soubani AO, Chen W, Jang H. The outcome of acute respiratory distress syndrome in relation to body mass index and diabetes mellitus. Heart Lung: J Acute Crit Care. 2015;44(5):441–7. 10.1016/j.hrtlng.2015.06.007 26212460

